# The Year 1902

**Published:** 1903-01

**Authors:** 


### EDITORIAL NOTES AND COMMENTS.

The Business office of THE JOURNAL-RECORD is No. 64 Marietta Street.

The Editorial office is 318-319-320 The Prudential.

Address all Business communications to Dr. M. B. Hutchins, Mgr.

Make remittances payable to THE ATLANTA JOURNAL-RECORD OF MEDICINE.

On matters pertaining to the Editorial and Original Communications address Dr. Bernard Wolff, Atlanta.

Reprints of original articles will be furnished at cost price. Requests for the same should always be made *on the manuscript*.

We will present, post-paid, on request, to each contributor of an original article, twenty (20) marked copies of THE JOURNAL-RECORD containing such article.

### THE YEAR 1902.

THE year that has just passed was not a notable one in the annals of medicine. It was signalized more by activity in sifting and elaborating than by discovery and invention. The problems which have vexed the medical mind for so long have been given more than usual attention. The subject of cancer has been pursued with unwonted diligence, though the net result can scarcely be regarded as an increment to our knowledge. There have been evidences of increased industry in organizing efforts to suppress tuberculosis, and if these efforts be crowned with even a modicum of success, the year that witnessed their inauguration will be rendered luminous in the history of medicine. The usual number of "cures" for tuberculosis have been exploited but have failed to impress the profession, and have one by one been abandoned. The study of that indeterminate quantity "immunity" has shown renewed popularity, but its elusive qualities have continued to baffle the attempts to explain it convincingly. As a corollary to this and as a more immediate result of the general prevalence of smallpox, the question of vaccination and re-vaccination has been much discussed, together with the relative merits of the different commercial forms of vaccine. The sum of opinion appears to be that the period of immunity conferred by vaccination is uncertain, and that re-vaccination should be employed as a preventive in times of exposure regardless of the date of former vaccination. This view does not of course throw any discredit upon the almost universally accredited efficiency of the procedure as a prophylactic.

The rôle of the mosquito as the intermediary host of certain types of communicable diseases has been more clearly established and elucidated, and this has led to practical results in the curtailment of these diseases.

In the department of therapeutics there have been some drugs of merit introduced which bid fair to fill a permanent place in the materia medica.

Probably the most noteworthy feature of therapeutic progress has been the confirmation of the efficacy of the X-ray as a therapeutic agent, especially in superficial malignant growths. The evidence is accumulating in many directions and there can be little doubt from the testimony of numerous observers that we possess in this agent a therapeutic resource of inestimable value. A respectable number of clinical facts concerning radiotherapy have been worked out by observers in this country, and material assistance has been rendered by them in establishing the value of the treatment.

The Finsen method of employing the chemical or ultra-violet rays of the arc light for therapeutic purposes, particularly in the diseases of the skin, has not received the attention that its claims seem to warrant. The expense of the mechanism and the slowness of results obtained by it have deterred many from availing themselves of it. But with cheapness and inevitable improvements it is likely that there will be much more attention given it in this country during the coming year. There has been a conspicuous revival of the static machine which had fallen into disuse. Apart from its use in connection with the X-ray, enthusiasts present its claims to recognition as an agent of great value and wide utility, but its exact status has not as yet been determined.

Perhaps the most notable achievement in the domain of surgery has been that of stripping the capsule of the kidney for the relief of various forms of nephritis—the so-called Edebohl's operation, from the name of its principal advocate. Cures of otherwise irremediable cases have been recorded, and the operation is being performed more and more by surgeons throughout the country.

Cocainization of the spinal cord, of which so much was said at the beginning of the past year, seems to have retired from notice, due perhaps to the fact that it proved to be uncertain and not less hazardous than the older methods of securing anesthesia. The surgical history of the year terminated in a blaze of glory with the arrival and wonderful exploits of Prof. Adolf Lorenz, of Vienna, a man of evident worth and of a modesty which successfully withstood the adulation and obsequious homage so lavishly displayed by his confrères in this country. He was a conquering hero, and, had he been a little younger and a little less gifted in the matter of whiskers, he might have inaugurated a kissing campaign which would have brought a flush of envy to the bronzed cheek of Hobson himself. The reaction after all this tumultuous applause and genuflexion (physiological) is awaited with interest.

The year just passed witnessed some gratifying concessions—to use a bland term—from the Christian Scientists to the medical profession. It will not be very much longer before this cult will be generally recognized as teaching what is merely a phase of suggestive therapeutics and its cherished idols be given over to the mammon of unrighteousness.

"Such are the changes that fleeting time procureth!"

The salient points of medical progress here briefly alluded to but faintly indicate the seething activity and fruitful unrest which lie beneath. The search is going on unceasingly, not only for that which we do not know, or, of which knowing only in part, we are prone in the arrogance of conceit to believe that the tale has not been told. It is fortunate that the spirit of complaisant self-sufficiency and content which characterizes the ignorant and slothful is counterpoised by that of a discontent which cherishes no illusions and seeks only for the absolute.

